# Measuring Collaboration Load With Pupillary Responses - Implications for the Design of Instructions in Task-Oriented HRI

**DOI:** 10.3389/fpsyg.2021.623657

**Published:** 2021-07-20

**Authors:** Dimosthenis Kontogiorgos, Joakim Gustafson

**Affiliations:** Division of Speech, Music and Hearing, Department of Intelligent Systems, KTH Royal Institute of Technology, Stockholm, Sweden

**Keywords:** social signal processing, pupillometry, dialogue and discourse, collaboration, common ground, least-collaborative-effort, situated interaction, referential communication

## Abstract

In face-to-face interaction, speakers establish common ground incrementally, the mutual belief of understanding. Instead of constructing “one-shot” complete utterances, speakers tend to package pieces of information in smaller fragments (what Clark calls “installments”). The aim of this paper was to investigate how speakers' fragmented construction of utterances affect the cognitive load of the conversational partners during utterance production and comprehension. In a collaborative furniture assembly, participants instructed each other how to build an IKEA stool. Pupil diameter was measured as an outcome of effort and cognitive processing in the collaborative task. Pupillometry data and eye-gaze behaviour indicated that more cognitive resources were required by speakers to construct fragmented rather than non-fragmented utterances. Such construction of utterances by audience design was associated with higher cognitive load for speakers. We also found that listeners' cognitive resources were decreased in each new speaker utterance, suggesting that speakers' efforts in the fragmented construction of utterances were successful to resolve ambiguities. The results indicated that speaking in fragments is beneficial for minimising collaboration load, however, adapting to listeners is a demanding task. We discuss implications for future empirical research on the design of task-oriented human-robot interactions, and how assistive social robots may benefit from the production of fragmented instructions.

## 1. Introduction

Interactive system designers need to better understand social conventions that people use in constructing utterances when structuring their speech in face-to-face interactions. It is particularly important to discriminate the fundamental discourse units that coordinate the incremental process of grounding. In that direction, researchers have examined grounding behaviour by considering utterance units given boundary signals such as prosodic boundaries and pauses (Traum and Heeman, 1996). Human utterances tend to be informal, contain disfluencies and often they are presented in fragments (Chai et al., 2014). In particular, Goodwin (1981) suggested that speakers tend to use coordination strategies such as fragmented production of utterances (*errors-in-production*), to coordinate turns with their listeners and facilitate the process of “co-production” of utterances. To achieve human-likeness, conversational interfaces may need to embrace such human properties of communication and should succeed in maintaining and coordinating these behaviours with human users.

By constructing “imperfect” human-like speech, conversational interfaces should consider how to produce utterances with cooperative and collaborative exchange of information (Uchida et al., 2019). Rational speakers will attempt to be cooperative and will plan to be as informative as it is required (Grice, 1989). In the Gricean view (Grice, 1957), speakers instruct listeners on their intentions by constructing indirect or incomplete requests in their references (Cohen, 1984). It seems that speakers construct utterances that are not straightforward, but potentially harder to comprehend and leaving the extra work of understanding up to the listeners (Davies, 2007). In compliance with the maxims of quantity, speakers withhold information from their listeners using the minimal effort required, and provide instructions using the *least-collaborative-effort* (Clark and Wilkes-Gibbs, 1986; Clark and Brennan, 1991; Brennan and Clark, 1996). As constructing minimal and simultaneously non-ambiguous utterances is “computationally expensive” (Davies, 2007), speakers may rely on listener cooperation to establish and maintain the process of grounding.

How people develop common understanding, based on the notion of “common ground”, has been studied by Clark and colleagues (Clark and Brennan, 1991), who have proposed the concept of *least-collaborative-effort*. In this principle, speakers maintain a fine-grained balance between effort and communication, and adapt their messages to their listeners' level of understanding^1^. As such, messages not appropriately adapted require more effort by both conversational partners and result in the exchange of more conversational turns to repair incomplete or confusing messages (Clark and Wilkes-Gibbs, 1986). Therefore, utterances are iteratively and collaboratively refined until common ground is established (Blaylock et al., 2003).

**Figure 1:**
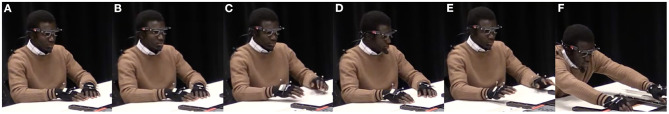
An illustration of the recurrent listener signals **(A–F)** to fragmented utterances (see dialogue sample 1).

In this iterative process, speakers do not always construct “one-shot” utterances, but tend to package pieces of information into *fragments*, or what Clark calls “installments” (Clark, 1996). These fragmentary discourse units are *contributions to common ground* and typically they are intonationally complete, suggesting the speaker is expanding turns until a satisfactory level of understanding has been reached. In this way, difficult or long utterances are presented in fragments, while the speaker continuously monitors the listener for understanding (Clark and Brennan, 1991). This is further illustrated in Figure 1 and Dialogue 1, a sample extracted from the assembly task corpus presented in this paper.

**Instructor:** [Utt-1] *So the first one you should take*. (Figure 1A)

**Builder:** *mhm* (Figure 1B)

**Instructor:** [Utt-1] *.is the frame*. (Figure 1B)

**Builder:** (looks at table, moves hands) (Figure 1C)

**Instructor:** [Utt-2] *But the one with the stripes*. (Figure 1D)

**Builder:** *okay* (Figure 1D)

**Instructor:** [Utt-3] *The black one*. (Figure 1D)

**Builder:** (looks toward the centre of the table) (Figure 1E)

**Builder:** (looks at object) (Figure 1E)

**Instructor:** [Utt-4] *With the stripes*. (Figure 1F)

**Builder:** (reaches for object) (Figure 1F)

**Dialogue 1**. A segment of *fragmented* task-oriented dialogue [interaction 20, piece 1]

**Instructor:** [Utt-1] *So we start with the largest piece with lines on it*

**Builder:** (looks at table, reaches for object)

**Dialogue 2**. A segment of *non-fragmented* task-oriented dialogue [interaction 32, piece 1]

As shown in transcript 1, the instructor gradually adds more turns and elaborates on the instructions incrementally. The listener verbally acknowledges receiving the information, nevertheless we cannot assume it is also understood. Producing the instruction in fragments allows the speaker to adapt and to repair or reformulate utterances in synchronisation to the listener's signals of understanding, therefore design their instructions as a “series of corrections” (Lindwall and Ekström, 2012). These instructional sequences are specifically addressed to the listener present, further demonstrating adaptive behaviour, a very challenging task for conversational interfaces (Rossi et al., 2017). However, how to identify these sequences and segment them into fragments is not straightforward.

According to Heeman (1999), speakers seem to segment their turns into “intonational phrases”, with variations in prosody and silent pauses. Researchers have modelled user responses to prosodic variations in grounding fragments spoken by a dialogue system (Skantze et al., 2006), indicating speakers' intonation in fragments also affects listener behaviour. These variations in intonation can signal a release of the speaker's turn (Traum and Hinkelman, 1992; Traum and Heeman, 1996), even if the speaker decides to expand the turn into more fragments, and where turn transitions are “relevant” (Sacks et al., 1978).

In this article, we look at fragmented utterances as a series of contributions to common ground. Particularly, we focus on how these utterances are constructed when humans produce instructions in task-oriented dialogues. We separate utterances to either: (a) *fragmented*, where the instruction is produced as a series of fragments (Dialogue 1), and (b) *non-fragmented*, where the whole instruction is produced in one utterance (Dialogue 2). To understand how fragmented instructions are produced, we examine how speakers' cognitive resources are allocated during utterance production by analysing the cognitive load of both conversational partners when either fragmented or non-fragmented utterances are constructed.

Cognitive load refers to the effort required in understanding and performing tasks (Sweller, 1988), and can be task-based, person-based, or based on coordination, typically referred to as collaboration load (Dillenbourg and Betrancourt, 2006). In collaboration load, speakers consider the mental effort of others and their actions, in order to predict their behaviour and take actions (Kolfschoten et al., 2012). Cognitive load has been measured in psycholinguistics utilising speech features such as pauses, articulation rate and disfluencies (Müller et al., 2001; Jameson et al., 2010; Womack et al., 2012). It has also been demonstrated that the more time a speaker takes to produce an utterance, the more cognitive resources are required (Schilperoord, 2002). The amount of silent pauses in particular seems to indicate “thinking” or increased cognitive load when constructing utterances (Chen et al., 2013).

Pupil dilation has also been found to correlate with cognitive load, hence in this study, we measure the conversational partners' pupil diameter as an indicator of cognitive load. Changes in pupil size reflect a diverse set of cognitive and affective states (Ahern and Beatty, 1979; Harrison et al., 2006; Kret and De Dreu, 2017) including arousal, interest, and effort. Pupil dilation is a highly sensitive measure of changes in cognitive resources and resource allocation. Several disciplines have used pupillometry as a response system to stimuli, and it has been an established method in psychophysiology^2^. Pupil dilations are involuntary reactions to stimuli, and research suggests that *task-evoked pupillary responses* provide an estimate of mental activity and task engagement (Gilzenrat et al., 2010; Laeng et al., 2012).

Task-evoked pupillary responses have also become popular in psycholinguistic research to measure language production and comprehension (van Rij et al., 2019). Researchers have studied mismatches between visual context, prosody and syntax (Engelhardt et al., 2010), speech rate (Koch and Janse, 2016), prosody in discourse processing (Zellin et al., 2011), and speech planning (Papesh and Goldinger, 2012). Chapman and Hallowell (2015) have observed larger pupillary responses during the comprehension of difficult words, compared to easier words. Grammatical complexity (Schluroff, 1982) and ambiguity in syntax (Ben-Nun, 1986; Schluroff et al., 1986) also seem to evoke larger pupil dilations. Also, larger pupillary responses have been observed in pragmatic manipulations, such as indirect requests when showing a figure of a closed window with the sentence “it is very hot here” (Tromp et al., 2016).

In sum, a large body of psycholinguistic research has examined speakers' cognitive resources in relation to language processing, yet many questions remain open on how cognitive load is allocated during grounding acts in face-to-face conversation. To our knowledge, this is the first investigation of how cognitive effort is mobilised within the principle of least-collaborative-effort. Whether speakers construct fragmented instructions due to the least-collaborative-effort, should likely be indicated in their measured cognitive load. There is evidence that pupil diameter is sensitive to sentence complexity and ambiguity, and in this article, our goal is to combine this prior knowledge and established methods from psychophysiology to examine the incremental production of instructions.

**Speaker effects**. Our first assumption is that *(1) speakers spend less cognitive resources in producing “one-shot” non-fragmented instructions, rather than fragmented instructions that are adapted to listeners*. One may expect that the extensive use of pauses and audience design contribute to higher cognitive load (Dillenbourg and Betrancourt, 2006), therefore more grounding effort for the conversational partners overall.
**Listener effects**. We also predict that *(2) speakers continuously attempt to reduce and minimise their listeners' cognitive load*. They do so by producing instructions in fragments, and therefore let the listener extract information incrementally.

## 2. Materials and Methods

Based on uncontrolled task-oriented and face-to-face dialogue, we examined how fragmented utterances affect speakers' pupillary responses and other interactional phenomena such as gaze behaviour, joint attention and mutual gaze.

### 2.1. Corpus

**Figure 2:**
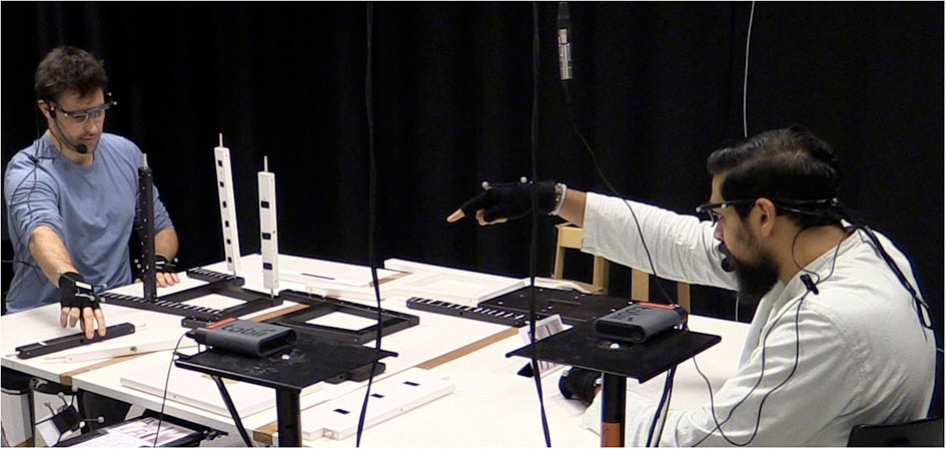
In the corpus analysed in this article human speakers instructed each other how to assemble an IKEA stool. Behavioural data was collected during the interactions.

Instructions play an important role in people's everyday tasks, from domestic to industrial domains. We have previously collected a corpus (Kontogiorgos et al., 2020) of task-oriented dialogue in which human speakers instruct each other how to assemble an IKEA stool (Figure 2). Using the concept of “Chinese Whispers,” an old children's game, subjects instructed the task to each other in a chain sequence. Participants first learned how to assemble the stool by an instructor, and in the next session they were told to take the role of the instructor and teach a new participant. That means, each participant did the task twice, first as a builder and then as an instructor. As such, instructors were not influenced by the experimenters on how to instruct, but only by previous instructors.

**Figure 3:**
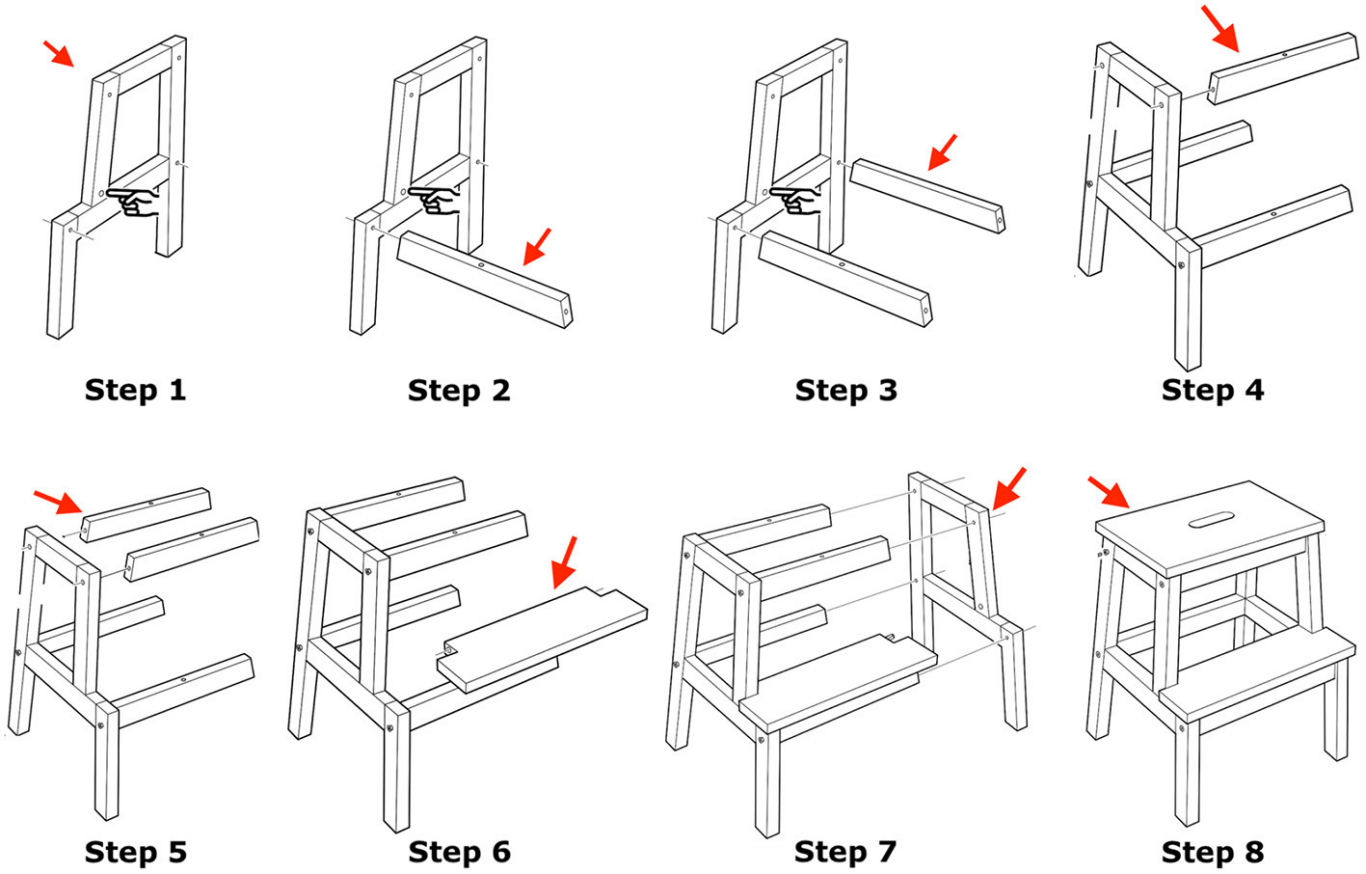
Subjects were given visual instructions in steps without verbal descriptors. The task is taken from the assembly procedure for the Bekväm stool sold by IKEA, circa 2018. In the steps visualised, the stool is assembled incrementally and secured with wooden bolts to make the assembly faster. *Images taken and adjusted from the IKEA instruction book (with permission)*.

Each instructor had to follow the same sequence of instructions, therefore at each step of the interaction we are aware of the speakers' intentions, however without controlling for how they will instruct. Instructors were given visual but not verbal instructions, as shown in Figure 3. As participants were instructing for the first time, they naturally engaged in collaborative and fragmented instructions, and uncontrolled situations of uncertainty.

Participants were seated across each other with a table between them that had a pile of all furniture pieces necessary for the assembly, including additional pieces for distraction. The furniture pieces used for stimuli had a variability in shape, sizes and colours, with black and white tape added to make them uniquely identifiable. The pieces were also placed in varying positions between the instructor and builder. Inevitably, it was up to each instructor how ambiguous their references would be and how many utterance reformulations would be necessary. The builders had not seen the assembled stool and did not know what it should look like. Using a chain effect of assembly, we had little control over how each task would be described. Participants had no time constraints as long as they would succeed in completing the task.

### 2.2. Participants

The corpus consists of 34 interactions, each one with an *instructor* and a *builder* sitting across each other. The mean age of the participants was 25.5 (SD 3.5). 11 reported female and 23 male, and the majority were students or researchers at KTH Royal Institute of Technology. All subjects were fluent in English, with a mean of 6.5 on a scale of 1–7 of self-reported English literacy. They reported little to no-interference of sensory equipment in the task (eye-tracking glasses, microphone, motion-capture gloves), with 2.2 (on a scale of 1–7) in an equipment interference questionnaire item. Participants also reported to be relatively experienced in assembling IKEA furniture (mean 4.6 on a scale of 1–7).

### 2.3. Procedure

First, participants filled an entry questionnaire with demographics, and signed a consent form for participation. Participants then entered the experiment room, they were introduced to their instructor and were guided to wear eye-tracking glasses, a microphone and motion-capture gloves, to capture their gaze, speech, and movements. The window blinds were closed and black curtains were surrounding the room. The room was illuminated with artificial light, always in the same conditions for all participants, and ensuring there is no interference in pupil measurements from external factors regardless the time of the day. Participants were instructed where to sit and how to wear the sensory equipment, however instructions given to participants regarding the task were minimal. In every interaction, the instructor was asked to instruct how to build the stool they had just built, while the builder was told to follow the instructor's guidance. The instructor (builder from previous interaction), was signalled to start the interaction and instruct (but without building) the assembly in steps. In each step, the instructor needed to instruct a new item that the builder was expected to assemble. After building (or instructing) the assembly task both subjects were asked to fill questionnaires in separate rooms, with items in engagement, task difficulty and measures of collaboration. Participants were rewarded with a cinema ticket (approximately 15 euro) at the end of the study, and each assembly task took 3.8 min on average. At the end of each interaction, the experimenters disassembled the stool and prepared the pieces for the next assembly.

### 2.4. Apparatus

A multi-sensory setup was used to capture multimodal signals from the conversation. An OptiTrack motion capture system was used to collect the positions of the furniture pieces, the participants' head position and orientation, as well as their hand gestures. Tobii mobile eye-trackers were used in combination with the motion capture system (Kontogiorgos et al., 2018) to extract participants' eye position in 3D space, as well as their pupil diameter. Motion-capture and eye-gaze data were extracted in 50 frames per second, and sensory calibration was required individually for each session. Additionally, close-talking microphones were used to collect channel-separated audio data. A clapperboard was used to indicate start and end times in all sensors, however sensory signals were also synced using a time server that provided timestamps for all events (Jonell et al., 2018). Finally, two video cameras from different angles were placed to record the interaction, used for qualitative analyses and annotations.

### 2.5. Analysis

As instructors in this corpus were engaged in a referential communication task, the conversations focused on objects and their identities. The purpose of the interaction was therefore to establish *referential identity*, “the mutual belief that the speakers have correctly identified a referent” (Clark and Brennan, 1991). This constrained nature of the task, allowed us to systematically examine linguistic choices from different speakers on the same objects. There are many alternatives a speaker may select to utter referring expressions, and multiple utterances may be co-produced in coordination with listeners (Goodwin, 1981; Clark and Wilkes-Gibbs, 1986).

#### 2.5.1. Annotations

The first part of the linguistic analysis was to tokenise each of the “pick-that-piece” instructions into utterance units that were annotated as being either non-fragmented or fragmented instructions. The *instructor* always initiated an instruction and the *builder* needed to understand the instructor's intent and assemble the objects as told. In some cases, instructions were grounded with a single utterance, but often instructions were fragmented into several utterance units and therefore grounding was delayed and satisfied incrementally (Schlangen et al., 2009). Using ELAN (Wittenburg et al., 2006) all instruction boundaries were then hand-segmented as being: (1) *intonationally complete*, or (2) having a *significant period of silence*. All fragments^3^ in the instructions were time-segmented (indicating the beginning and end of each fragment). The end of each fragment can be regarded as a *transition relevance place (TRPs)*, i.e., moments in the conversation where the listener could have taken the turn, regardless if the turn actually switched (Sacks et al., 1978). Even though the instructions are segmented into fragmented utterances for the sake of the analysis, they comprise a single instruction unit (on one referent object), defined by the visual guide given to the instructors (Figure 3). The utterances were then coded by sequence^4^ [Utt-1, Utt-2,…, Utt-n] as shown in the dialogue sample above^5^, and they typically repair previous content of the instruction unit or expand the speaker's turn with additional information.

#### 2.5.2. Data

Using the time-tagged annotations of the instructor utterances in ELAN, gaze and motion data from both the instructor and the builder could be coupled to each segment in the spoken instructions. Due to sensor errors, six interactions were excluded from the analysis. From the remaining 28 interactions, a total of 263 instruction units were extracted^6^, in which 118 were *non-fragmented* instructions (with a single utterance) and 145 were *fragmented* instructions (with several utterances). In these 145 *fragmented* instructions, there were a total of 359 utterances. The *fragmented* utterances were then split by sequence, leading to 140 instructions with at least 1 fragment (Utt-1), 137 with at least 2 fragments (Utt-2), 54 with at least 3 fragments (Utt-3), and 23 with 4 fragments (Utt-4)^7^. These fragmented utterances typically labelled taxonomically the objects in the shared space between the instructor and the builder using object properties such as shape, size, position and colour.

### 2.6. Behavioural Measures

Using the aforementioned segmentations of the utterances, temporal multimodal data was extracted. All measures extracted in this study are based on automatic annotation of behavioural data. Due to the spontaneous nature of conversation in this corpus, each instructor utterance is treated as a stimulus, to which behavioural responses occur—either by the one producing it, or the one trying to comprehend it. Several instructions and utterances are extracted from each interaction. In a subsequent analysis, the instructions that are produced in fragments are investigated in isolation in order to examine the progression of the measures as grounding is incrementally established.

#### 2.6.1. Eye-Gaze

The sensory data, with a frequency of 50 fps, from motion-capture and the eye-tracking glasses were combined using a single coordinate system to calculate metrics that estimate the conversational partners' visual attention in the interactions—measures that were also used in work from Kontogiorgos et al. (2019). The most prominent visual target during each instructor utterance was estimated using the visual angle toward each object and toward the conversational partner by using majority voting (i.e., the target with the maximal proportional gaze). The collected eye-gaze data can produce missing sample segments due to the jerky movement of the eyes or eye blinks occluding the infrared cameras of the eye-trackers. This resulted in a 77% rate of valid gaze data points for the instructor and 78% rate of valid gaze data points for the builder in this dataset.

Typically gaze signals are aligned with verbal utterances, which are important to disambiguate confusing or incomplete referring language (Meyer et al., 1998). The timings for utterances were derived by the ELAN utterance segmentations. Each instruction segment was concerned with a specific object target to which both instructor and builder should be attending to with their gaze (by task design). The following eye-gaze metrics were calculated for both the Instructor and Builder. The measures reflect the proportional amounts of gaze toward the instructor's referent target object, and a measure of how much time it takes for the conversational partners to gaze at the target referent object.

For each instructor utterance segment the **First Gaze to Target Referent** is computed. This measure represents the time (in seconds) it took the Builder and the Instructor to identify and gaze at the referent object (counting from the beginning of the instructor's utterance).
**Gaze to Target Referent:** the proportion of gaze directed to the referent object during each utterance was computed. Less gaze toward the referent object can reflect more gaze toward the distractor objects (scanning of the visual scene) or toward the conversational partner.
**Gaze to Other Objects:** this measure reflects the proportional gaze to other distractor objects and not the referent object.
**Gaze to Person:** the proportion of gaze toward the conversational partner during each segment of the instruction. This measure represents gaze that is directed toward the conversational partner and not toward the referent or the distractor objects, as it is important to establish understanding in these grounding sequences of instructions.
**Joint Attention:** the proportion of time in the utterance where both individuals are following each other's gaze (toward the same referent object the instructor has uttered, and at the same time).
**Mutual Gaze:** the proportion of time in the segment where the conversational partners are looking at each other (both partners' Gaze To Person measure has to be directed at each other, and at the same time).

#### 2.6.2. Pupillary Responses

**Figure 4:**
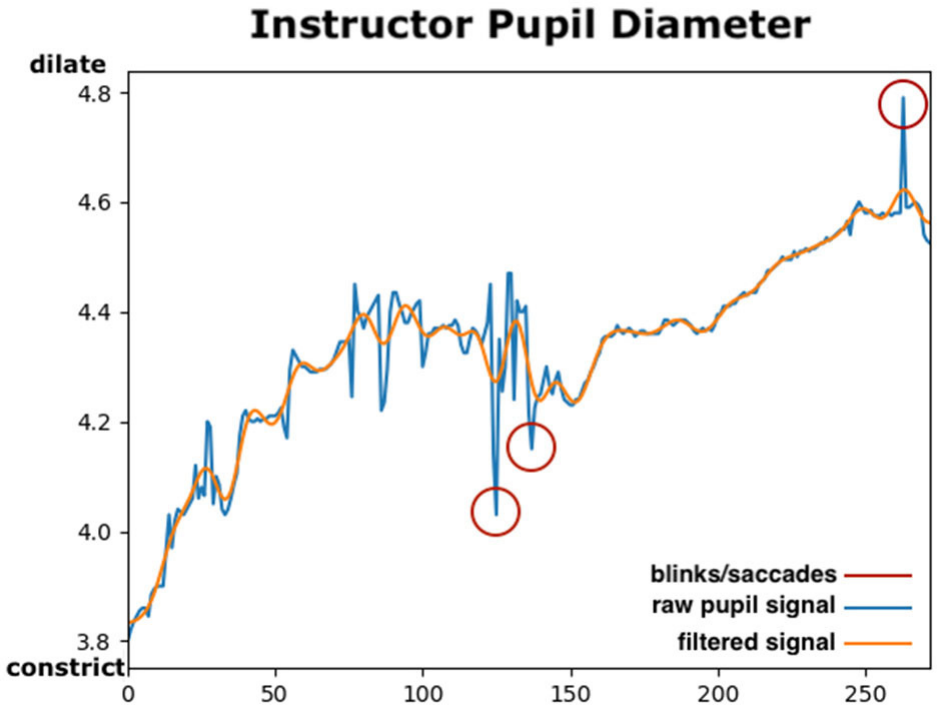
The mean pupil diameter in a segment of Instructor 3. The window represents changes in pupil diameter size in about 5 s between two utterances. The blue line depicts the raw sensory data, and the orange line shows the filtered signal using a low-pass filter. Potential eye-blinks/saccades are shown in red circles and how the filter adjusts the resulted pupil signal. *X*-axis indicates the pupil diameter size in mm and *y*-axis indicates time in frames (50 Hz). This figure is best viewed in colour.

The Tobii eye tracker provided a continuous pupil signal with a frequency of 50 Hz, i.e., the same as the eye movements measures described above. Using each instructor utterance as the stimulus, pupil diameter size data could be extracted during each verbal instruction segment that had been annotated in ELAN. Pupillary data requires some pre-processing before analysing each of the segments individually (Kret and Sjak-Shie, 2019). First the absolute mean pupil diameter size variable was computed. Left and right eye pupil diameters are highly correlated, and if pupil data from both the left and the right eye was available, their mean value was calculated, otherwise the data from the eye with available data was used. Since the sensory pupil data includes a lot of noise and jittering, a filter to smooth the signal and interpolate missing values was generated. In Figure 4, a sample of the raw and filtered pupil diameter signals is presented. Using the SciPy library (Virtanen et al., 2020), a 5th order low-pass Butterworth filter^8^ with a 4 Hz cut-off frequency was applied to smooth the pupil diameter data. Using the filter, artefacts causing sudden changes in the pupil such as eye blinks, saccades or sensor noise were removed and interpolated.

After noisy samples have been filtered, outliers may still remain in the data, especially in small utterance segments. To control for all remaining outliers, the z-score for all pupil data was computed and samples were excluded for which the pupil diameter was estimated to be more than two standard deviations away of the average pupil diameter size, for example potential samples outside the feasible range [1.5–9 mm] (Kret et al., 2014). Participant variability in the samples was further taken into account in the statistical analysis, by including the participant ID as a random intercept in the statistical model as explained in the next section.

In the beginning of each interaction, the baseline of the pupil diameter was extracted for a few seconds for both subjects before the instruction stimuli. Changes in the pupil diameter thereafter were computed in comparison to the baselines, in order to ensure that dilations in the pupil are due to participants engaging in the instructions and task stimuli rather than other factors. The following metrics were calculated for both subjects during the instruction utterances.

**Mean Pupil Dilation:** the mean value of the pupil dilation (difference compared to baseline) during the utterance segment. This measure reflects dilation on positive values and contraction on negative values.
**Peak Pupil Dilation:** in this measure the maximum dilation (difference compared to baseline) during the instruction segment was computed.
**Pupil Diameter Slope:** a linear regression was applied on the pupil diameter size in each utterance segment to compute the steepness in the increase in pupil dilation.

#### 2.6.3. Time

Finally, using the aforementioned utterance segmentations from ELAN, each utterance duration in seconds was calculated. While utterance duration differs among instructions, as they are produced by subjects, behavioural measures extracted are proportional, therefore normalised across utterances.

## 3. Results

Using the behavioural metrics described and the separation of instruction units into *fragmented* and *non-fragmented*, we present a systematic analysis on: (1) how the two types of instructions differ in eye-gaze behaviour and pupillary responses, and (2) how the utterance sequence of the fragments progress to incremental grounding. For the analysis of (1), we compare the utterance measures per subject and conduct pairwise comparisons on fragmented and non-fragmented utterances. For (2) we analyse the fragmented utterances in isolation (Utt1-Utt4) and examine how each new fragment affects the behavioural measures. In both cases, multiple utterances per instructor were compared.

Analyses were conducted in R (R Core Team, 2020) and Linear Mixed-Effects Models (LMM) were used with the *lme4* package (Bates et al., 2015), controlling for the variance of each interaction and each instructed object as random intercepts. For (1) the utterance type (with two factors) was used as fixed factor, and for (2) the sequence of the fragment (with four factors) was used as fixed factor. In both (1) and (2) the role of the participant in the interaction (Builder or Instructor) was also added as a fixed factor. For both analyses we present the chi-square and *p*-values derived from maximum likelihood estimation tests, comparing the given model to intercept-only models. The interaction of the predictors (Fragment*Role) was also tested. Finally, a correlation analysis was conducted to determine if there is a linear association of the number of fragments spoken by instructors with any of the behavioural measures.

### 3.1. How Do Fragmented and Non-fragmented Instructions Differ?

#### 3.1.1. Eye-Gaze

**Figure 5:**
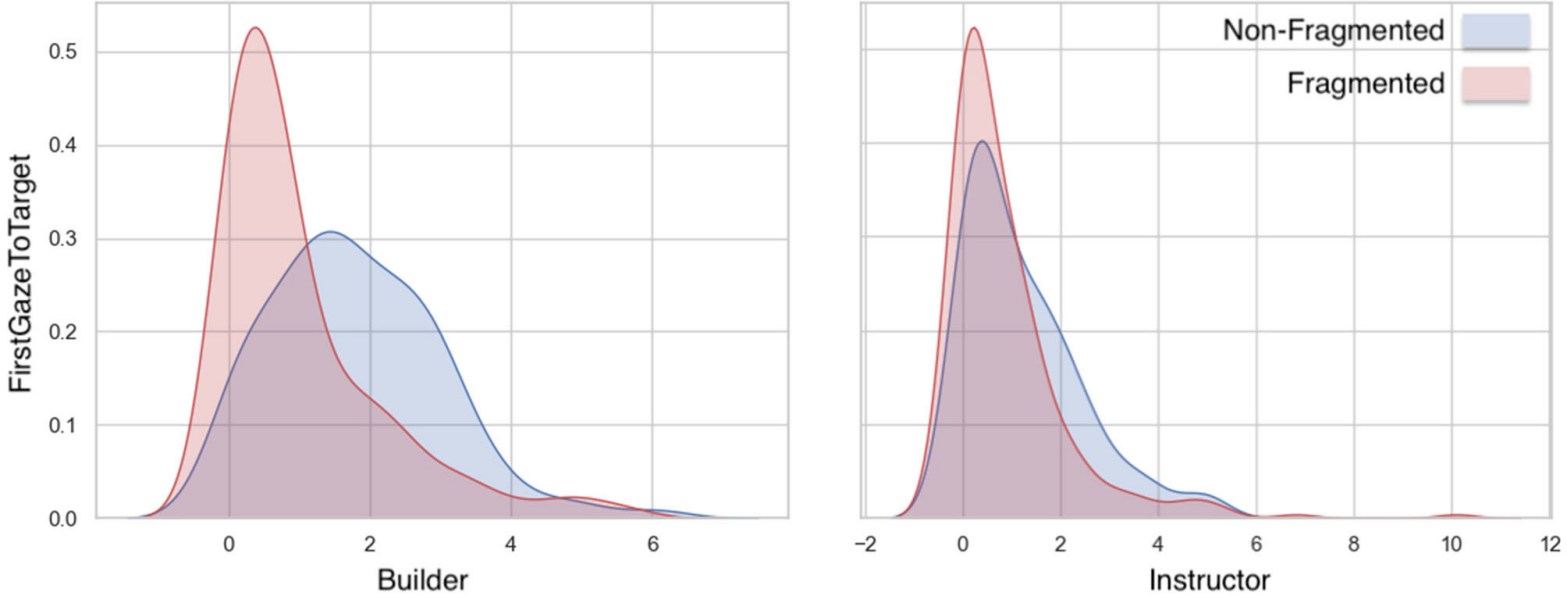
Differences in variance of FirstGazeToTarget measure among fragmented and non-fragmented instructions.

**Figure 6:**
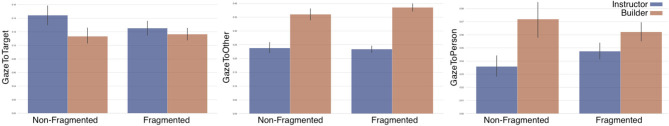
Differences in eye-gaze measures among fragmented and non-fragmented instructions: **(A)** Proportional gaze to target object, **(B)** Proportional gaze to other objects, and **(C)** Proportional gaze to person. Error bars indicate the standard errors of the means.

**First Gaze to Target:** Linear Mixed-Effects Model analyses (Figure 5 and Table 1) showed significant effects in the factors of Fragment and Role, as well as on their interaction. Builders were faster at identifying referents in fragmented utterances than in non-fragmented utterances.
**Gaze to Target:** LMM analyses (Figure 6 and Table 1) showed no significant differences in builders' or instructors' gaze to target referent among fragmented and non-fragmented instructions.
**Gaze to Other:** Significant differences were found in the proportional amount of gaze to other (distractor) objects in the Role factor. As expected, Builders gazed more at distractor objects than Instructors. A significant interaction was also found between the Fragment and Role factors. No significant effect was found on the factor of Fragment in isolation.
**Gaze to Person:** Similarly, no significant differences were found in gaze to person among fragmented and non-fragmented instructions. Significant effects were found however in the factor of Role and in the interaction among the two factors.
**Joint Attention:** LMMs showed no significant main effect in joint attention among fragmented and non-fragmented instructions in joint attention among the conversational partners (Table 2).
**Mutual Gaze:** Similarly, no significant differences were found in mutual gaze in the conversational partners among fragmented and non-fragmented instructions.

**Table 1:** LMM on gaze features of the Builder [B] and the Instructor [I], in Fragment (NoF-F) and Role (B-I) fixed factors.

**Predictor**	**[B] Non-fragmented**	**[B] Fragmented**	**[I] Non-fragmented**	**[I] Fragmented**	**Fragment χ^2^**	***p*-value**	**Role χ^2^**	***p*-value**	Fragment ^*^ Role χ^2^	***p*-value**
FirstGazeToTarget	1.79 ± 1.19	1.02 ± 1.18	1.22 ± 1.20	0.88 ± 1.28	27.32	^***^	6.64	^**^	39.93	^***^
GazeToTarget	0.11 ± 0.13	0.11 ± 0.17	0.14 ± 0.16	0.12 ± 0.19	0.05		1.93		2.87	
GazeToOther	0.36 ± 0.23	0.38 ± 0.26	0.23 ± 0.21	0.23 ± 0.22	0.49		92.24	^***^	93.54	^***^
GazeToPerson	0.07 ± 0.15	0.06 ± 0.14	0.03 ± 0.09	0.04 ± 0.11	0.43		6.75	^**^	8.65	^*^

*P-value indicators*:

* *p ≤ 0.05*,

** *p ≤ 0.01*,

*** *p ≤ 0.001*.

**Table 2:** LMM on group attention features in Fragment (NoF-F) fixed factor.

**Predictor**	**Non-fragmented**	**Fragmented**	**Fragment χ^2^**	***p*-value**
Joint Attention	0.02 ± 0.03	0.03 ± 0.07	3.42	
Mutual Gaze	0.001 ± 0.006	0.002 ± 0.017	0.57	

P-value indicators: *p ≤ 0.05, **p ≤ 0.01, ***p ≤ 0.001.

#### 3.1.2. Pupillary Responses

**Figure 7:**
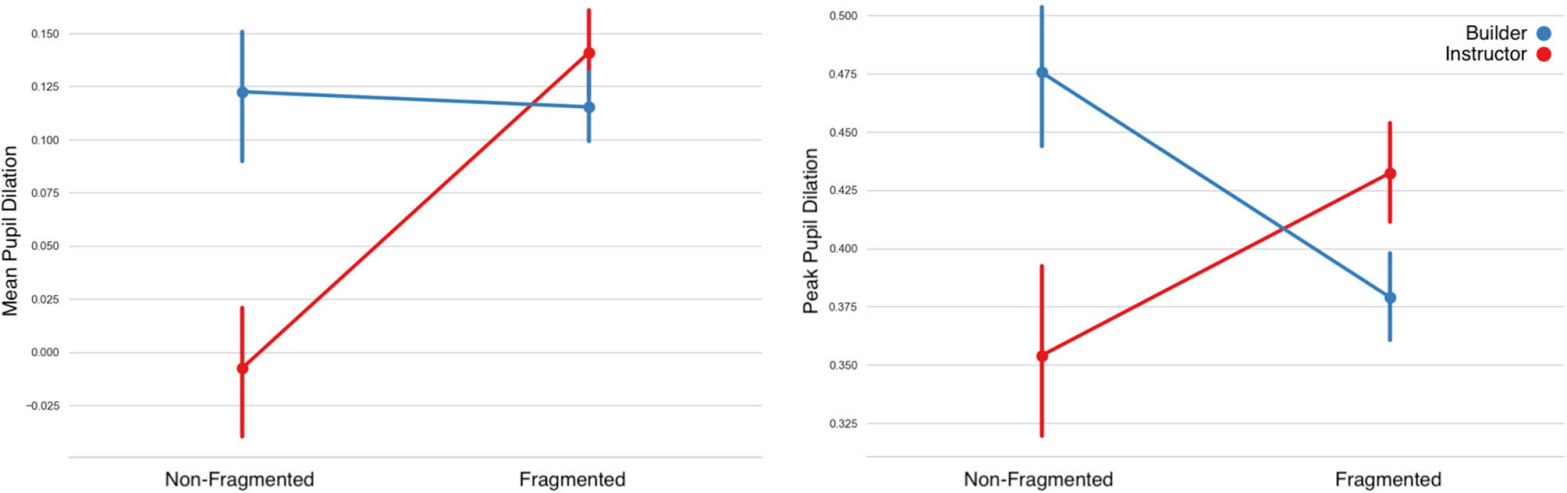
Changes in pupil diameter during the Instructor's utterances separated by Non-Fragmented/Fragmented Instruction class. Instructors' cognitive load appears to be higher in constructing fragmented instructions overall, while the opposite effect is found for builders. Error bars indicate standard error of the means.

**Mean Pupil Dilation:** LMM (Figure 7 and Table 3) showed no significant differences on the factors of Fragment and Role. A significant difference however was found in the interaction of the two factors, Instructors' pupil dilation was higher in fragmented instructions.
**Peak Pupil Dilation:** Similarly, no significant effect was found on the Role factor, however a significant effect was found on the Fragment factor and on the interactions of the Fragment and Role factors.
**Pupil Diameter Slope:** LMM showed no significant main effect on the factor of Fragment, however significant effects were found on the factor of Role and on the interaction between the two factors. Instructors' mean pupil diameter increased more in fragmented utterances.

**Table 3:** LMM on pupil diameter features of the Builder [B] and the Instructor [I], in Fragment (NoF-F) and Role (B-I) fixed factors.

**Predictor**	**[B] Non-fragmented**	**[B] Fragmented**	**[I] Non-fragmented**	**[I] Fragmented**	**Fragment χ^2^**	***p*-value**	**Role χ^2^**	***p*-value**	Fragment ^*^ Role χ^2^	***p*-value**
Mean Dilation	0.12 ± 0.33	0.11 ± 0.30	0.00 ± 0.33	0.14 ± 0.35	0.44		0.41		14.96	^**^
Peak Dilation	0.47 ± 0.33	0.37 ± 0.34	0.35 ± 0.37	0.43 ± 0.39	7.61	^**^	0.30		22.26	^***^
Slope	0.0002 ± 0.0026	0.0002 ± 0.0041	0.0009 ± 0.0029	0.0012 ± 0.0048	0.27		10.48	^**^	10.85	^*^

*P-value indicators*:

* *p ≤ 0.05*,

** *p ≤ 0.01*,

*** *p ≤ 0.001*.

#### 3.1.3. Time

**Figure 8:**
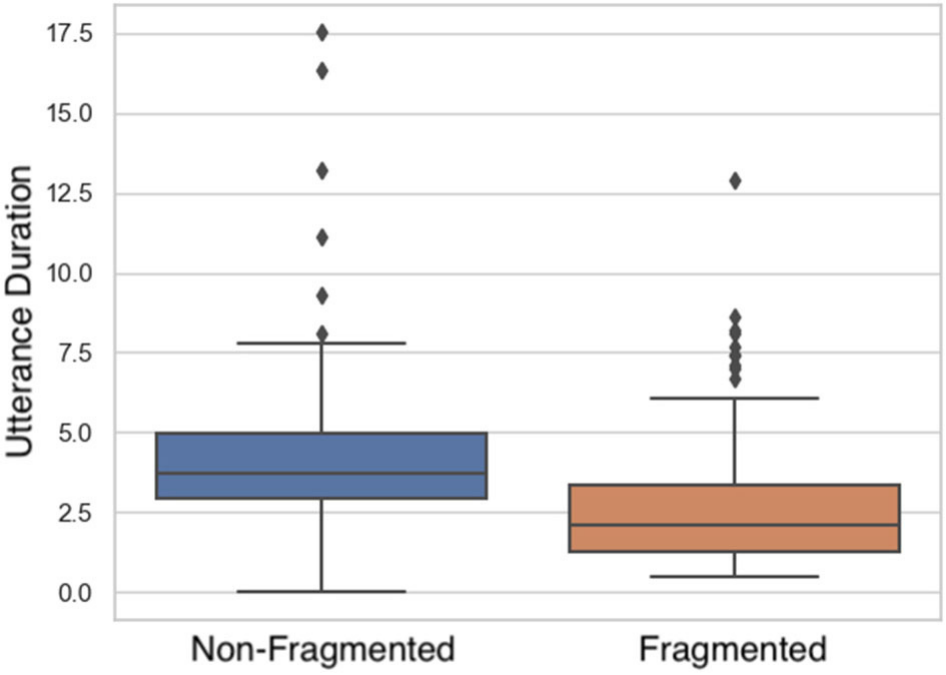
Differences in utterance duration (in seconds) among non-fragmented and fragmented instructions.

**Duration:** LMM analyses (Figure 8 and Table 4) showed a significant main effect in utterance duration among fragmented and non-fragmented instructions. Non-fragmented utterances were longer in time than fragmented utterances.

**Table 4:** LMM on utterance duration in Fragment (NoF-F) fixed factor.

**Predictor**	**Non-fragmented**	**Fragmented**	**Fragment χ^2^**	***p*-value**
Duration	4.24 ± 2.49	2.49 ± 1.65	75.71	^***^

P-value indicators: *p ≤ 0.05, **p ≤ 0.01,

*** *p ≤ 0.001*.

### 3.2. Effects of Utterance Sequence in Fragmented Instructions

We further analysed how behavioural measures in fragmented utterances progress incrementally within the same instruction unit and how the sequence of the fragment affects eye-gaze and pupillary responses among the conversational partners. In order to investigate the progression of the measures for each fragmented instruction, fragmented utterances were examined in isolation and the sequence of utterances were compared as fixed factors in the statistical analysis (Utt1-Utt2-Utt3-Utt4). Here all the fragmented instruction utterances are used, and the non-fragmented instructions are discarded. Interaction ID and instruction steps are also used in this analysis as random intercepts.

#### 3.2.1. Eye-Gaze

**Figure 9:**
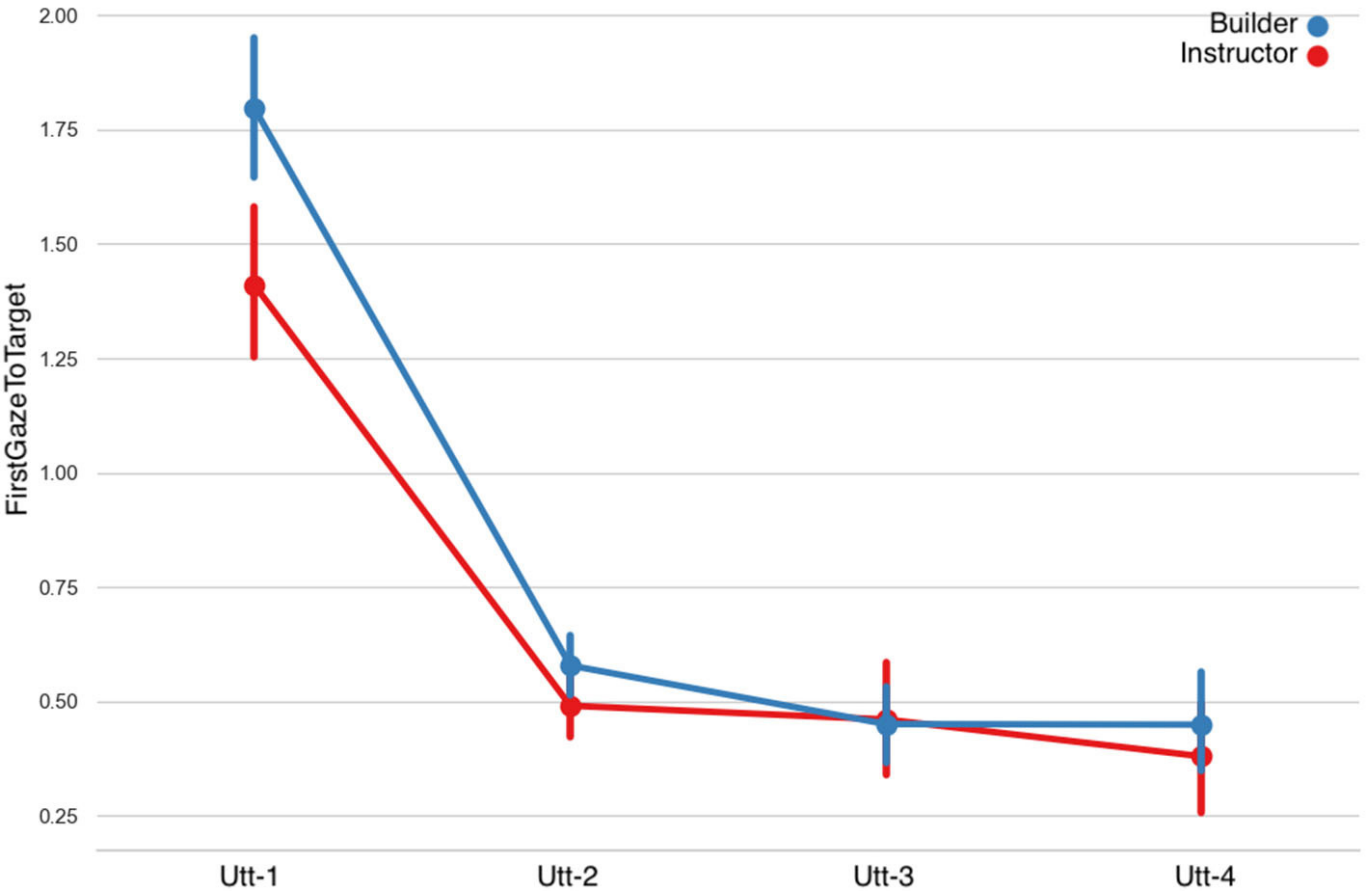
First gaze to target object per utterance sequence. Error bars indicate standard error of the means.

**Figure 10:**
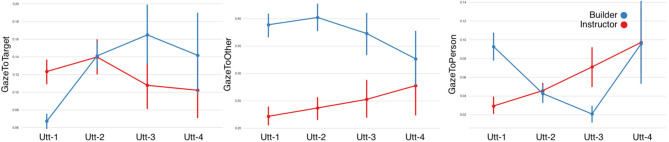
Eye-gaze features per utterance sequence. **(A)** GazeToTarget referent, **(B)** GazeToOther referents, and **(C)** GazeToPerson. Error bars indicate standard error of the means.

**Figure 11:**
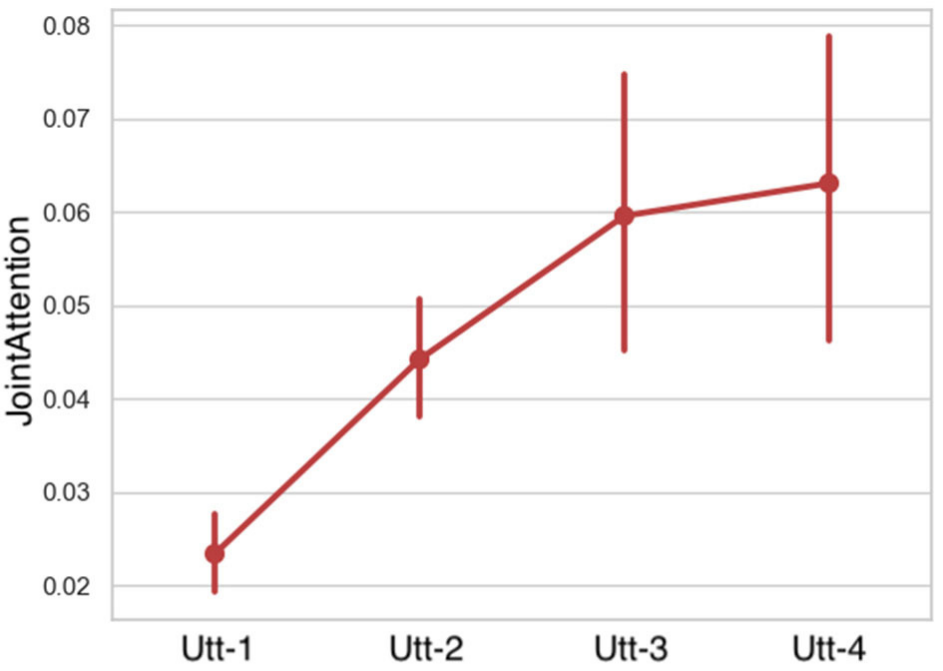
Joint attention among the conversational partners per utterance sequence. Error bars indicate standard error of the means.

**First Gaze to Target:** LMMs showed that the first gaze to the target object as a predictor caused a significant main effect on the Fragment factor, however no significant differences were found in the Role factor. A statistically significant interaction effect was also observed (Figure 9 and Table 5). The duration of the builders' and instructors' first gaze to the target decreased in time.
**Gaze to Target:** fitting LMMs on the fixed factors of fragment-sequence and participant-role showed that gaze to the target object caused a significant main effect on the fragment factor however no significant differences were found on the role factor (Figure 10 and Table 5). A statistically significant interaction was observed among the fragment-sequence and participant-role factors.
**Gaze to Other:** Regarding the gaze to other objects predictor, LMMs showed no significant differences among the Fragmented factor, however a significant effect was found on the Role factor, as well as an interaction effect was observed.
**Gaze to Person:** LMMs on the fixed factors of fragment-sequence and participant-role showed no significant effects. A statistically significant interaction effect was found however between the two factors.
**Joint Attention:** LMMs showed that the conversational partners' joint attention caused a significant main effect on the Fragment factor. Joint attention increased in time (Figure 11 and Table 6).
**Mutual Gaze:** LMMs showed that mutual gaze caused no significant differences on the Fragment factor among the conversational partners.

**Table 5:** LMM on gaze features of the Builder [B] and the Instructor [I], in Fragment (Utt1-Utt4) and Role (B-I) fixed factors.

**Predictor**	**[B] Utt-1**	**[B] Utt-2**	**[B] Utt-3**	**[B] Utt-4**	**[I] Utt-1**	**[I] Utt-2**	**[I] Utt-3**	**[I] Utt-4**	**Fragment χ^2^**	***p*-value**	**Role χ^2^**	**p-value**	Fragment ^*^ Role χ^2^	***p*-value**
FirstGazeToTarget	1.79 ± 1.45	0.57 ± 0.60	0.45 ± 0.48	0.44 ± 0.43	1.40 ± 1.64	0.49 ± 0.65	0.46 ± 0.65	0.37 ± 0.44	101.42	^***^	1.33		107.25	^***^
GazeToTarget	0.06 ± 0.10	0.14 ± 0.17	0.16 ± 0.25	0.14 ± 0.22	0.12 ± 0.16	0.13 ± 0.23	0.10 ± 0.18	0.10 ± 0.15	13.22	^**^	0.78		25.50	^***^
GazeToOther	0.38 ± 0.24	0.40 ± 0.28	0.37 ± 0.28	0.32 ± 0.25	0.21 ± 0.20	0.23 ± 0.23	0.25 ± 0.25	0.27 ± 0.26	0.66		71.21	^***^	75.09	^***^
GazeToPerson	0.09 ± 0.17	0.04 ± 0.11	0.02 ± 0.06	0.09 ± 0.20	0.02 ± 0.10	0.04 ± 0.09	0.07 ± 0.15	0.09 ± 0.17	5.81		3.25		29.98	^***^

*P-value indicators*:

* *p ≤ 0.05*,

** *p ≤ 0.01*,

*** *p ≤ 0.001*.

**Table 6:** LMM on group attention features in Fragment (Utt1-Utt4) fixed factor.

**Predictor**	**Utt-1**	**Utt-2**	**Utt-3**	**Utt-4**	**Fragment χ^2^**	***p*-value**
Joint Attention	0.02 ± 0.05	0.04 ± 0.07	0.05 ± 0.11	0.06 ± 0.08	14.38	^**^
Mutual Gaze	0.002 ± 0.017	0.001 ± 0.014	0.002 ± 0.020	0.004 ± 0.022	0.55	

P-value indicators: *p ≤ 0.05,

** *p ≤ 0.01*,

***p ≤ 0.001.

#### 3.2.2. Pupillary Responses

**Figure 12:**
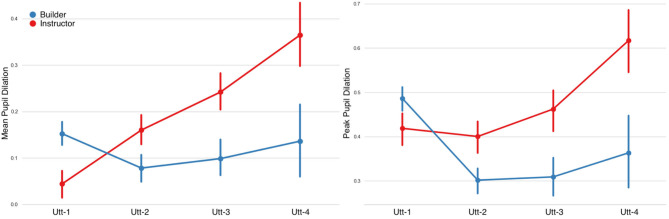
Pupil dilation (in mm) per utterance sequence. Mean Pupil Dilation on the left and Peak Pupil Dilation on the right. An interaction effect can be observed in both measures. Error bars indicate standard error of the means.

**Mean Pupil Dilation:** fitting LMMs on the fixed factors of fragment-sequence and participant-role showed a significant main effect on the fragment-sequence factor, however no significant differences were found on the participant-role factor. A statistically significant effect was also observed in the interaction between the two factors (Figure 12 and Table 7).
**Peak Pupil Dilation:** LMM analyses showed significant main effects on the fragment-sequence factor, on the role factor, as well as on their interaction.
**Pupil Diameter Slope:** LMMs indicated no significant main effects on the fragment-sequence factor, however a statistically significant effect was observed on the participant-role factor.

**Table 7:** LMM on pupil diameter features of the Builder [B] and the Instructor [I], in Fragment (Utt1-Utt4) and Role (B-I) fixed factors.

**Predictor**	**[B] Utt-1**	**[B] Utt-2**	**[B] Utt-3**	**[B] Utt-4**	**[I] Utt-1**	**[I] Utt-2**	**[I] Utt-3**	**[I] Utt-4**	**Fragmentχ^2^**	***p*-value**	**Role χ^2^**	***p*-value**	Fragment ^*^ Role χ^2^	***p*-value**
Mean Dilation	0.15 ± 0.29	0.07 ± 0.31	0.09 ± 0.28	0.13 ± 0.38	0.04 ± 0.34	0.16 ± 0.36	0.24 ± 0.28	0.36 ± 0.33	10.20	^*^	1.55		41.50	^***^
Peak Dilation	0.48 ± 0.31	0.30 ± 0.33	0.30 ± 0.31	0.36 ± 0.42	0.41 ± 0.40	0.40 ± 0.42	0.46 ± 0.34	0.61 ± 0.35	16.68	^***^	4.90	^*^	40.94	^***^
Slope	0.0001 ± 0.0026	0.0001 ± 0.0050	0.0002 ± 0.0049	0.0018 ± 0.0037	0.0006 ± 0.0030	0.0015 ± 0.0052	0.0010 ± 0.0067	0.0019 ± 0.0055	4.69		6.79	^**^	13.25	

*P-value indicators*:

* *p ≤ 0.05*,

** *p ≤ 0.01*,

*** *p ≤ 0.001*.

#### 3.2.3. Time

**Figure 13:**
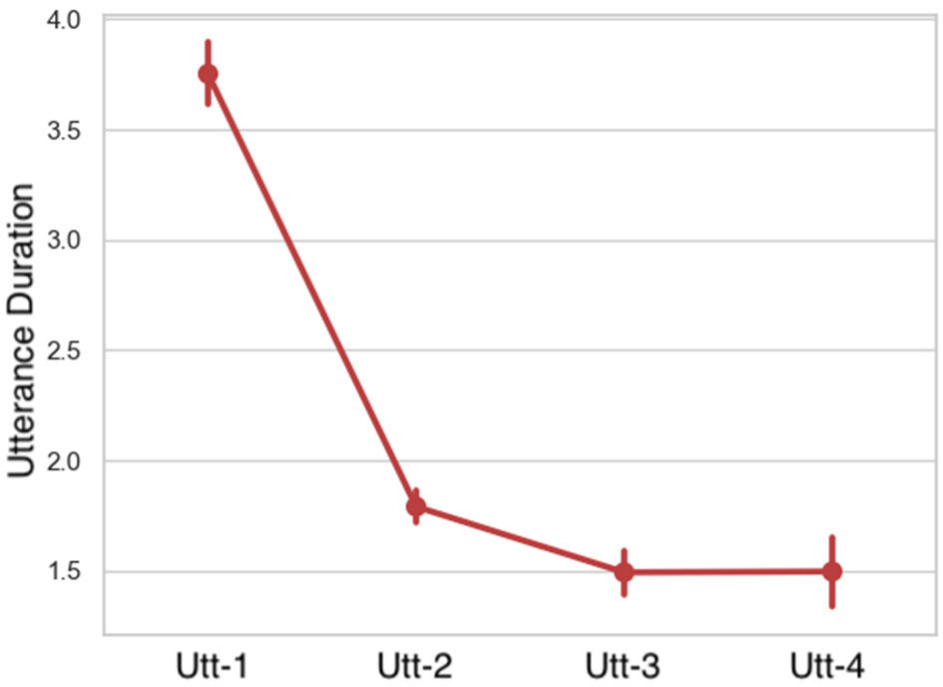
Duration per utterance sequence. Error bars indicate standard error of the means.

**Duration:** comparing the duration in seconds of each utterance, LMMs indicated that utterance duration caused a significant main effect. Duration of the utterances incrementally decreased (Figure 13 and Table 8).

**Table 8:** LMM on utterance duration in Fragment (Utt1-Utt4) fixed factor.

**Predictor**	**Utt-1**	**Utt-2**	**Utt-3**	**Utt-4**	**Fragment χ^2^**	***p*-value**
Duration	3.75 ± 1.80	1.79 ± 0.89	1.49 ± 0.73	1.49 ± 0.78	182.09	^***^

P-value indicators: *p ≤ 0.05, **p ≤ 0.01,

*** *p ≤ 0.001*.

#### 3.2.4. Correlation Analysis

Using the behavioural measures described above, an exploratory correlation analysis was conducted that evaluated how the metrics correlate with the number of fragments used in each instruction. Spearman's correlation coefficients were calculated between **gaze**, **pupil**, and **time** measures with the **number of fragments** per instruction, as some speakers tend to instruct using more fragments than others. A Bonferroni correction was applied to adjust for multiple comparisons, and only correlation coefficients larger than 0.15 are reported.

A number of gaze behaviour characteristics were associated with fragmented utterance production. The more fragmented utterances in instructions spoken the faster were Builders (FirstGazeToTarget: *r* = –0.47, *p* < 0.001) and Instructors (FirstGazeToTarget: *r* = –0.36, *p* < 0.001) at identifying the target referent object as more information is incrementally available by Instructors prior to every new fragment. More fragments were also negatively associated with Builders' gaze to the Instructor (GazeToPerson: *r* = –0.24, *p* < 0.001), potentially indicating more gaze toward the assembly task. Additionally, more fragments were negatively associated with their duration (Duration: *r* = –0.61, *p* < 0.001), as indicated in prior analysis in section 3.2.3 and suggesting each new fragment may have less information as instructors spoke shorter utterances. Finally, the number of fragments correlated with the Mean Pupil Dilation of the Instructor (MeanPupilDilation: *r* = 0.26, *p* < 0.001), indicating an increase in cognitive load for Instructors the more fragments they spoke. On the contrary, more fragments were negatively associated with Builders' Peak Pupil Dilation (PeakPupilDilation: *r* = –0.21, *p* < 0.001), indicating a decrease in cognitive load for builders as more fragments are spoken by instructors. There were no other statistically significant correlations observed on the behavioural cues explored.

## 4. Discussion

In this article we examined fragmented utterances as grounding acts in relation to speakers' cognitive effort, and what strategies speakers use to construct instructions in fragments. We utilised pupillary responses to instructions along with eye-gaze behaviour. We found that new speaker contributions to common ground caused an increase in speakers' cognitive load in order to reduce listener uncertainty and maintain mutual understanding.

The “Chinese Whispers” paradigm we utilised encouraged collaborative and fragmented production of instructions, while maintaining constraints on how the task would progress. This means, the corpus used in this analysis was of task-oriented nature, therefore the discourse progressed by establishing referential identities and task actions. While both builder and instructor attempted to achieve a common goal, it was the instructor's responsibility to resolve uncertainty and misunderstanding by having complete knowledge of the task. The multisensory infrastructure we used to capture speaker behaviour provided a multimodal understanding of the interactions, including information speakers do not have access to in conversation (i.e., variations in pupil diameter). These results give a first indication on how speakers mobilise cognitive resources in grounding behaviours and within the principles of least-collaborative-effort.

Our main goal was to identify whether behavioural and pupillary responses differ among non-fragmented and fragmented utterance sequences. The behavioural data indicated that listeners' cognitive load is decreased when speakers provide more information on the task, while speakers' cognitive resources seem to increase. Our assumption is that speakers continuously reformulate their utterances to reduce listener uncertainty and maintain mutual understanding, however adapting to the listener is a demanding task. This episodic production of instructions contributes to minimising the collaborative effort, rather than minimising the speakers' own effort (Chai et al., 2014; Fang et al., 2014). In light of these results, we discuss how speaker and listener behaviour affect the construction of fragmented instructions and how can research in conversational interfaces design instructions adapted to users.

### 4.1. Effects of Speaker Behaviour

During the task, both the instructor and the builder contributed to establish mutual understanding. However, it was the instructor that had complete knowledge of the task. Every instructor message can be viewed as an “information unit” (Halliday, 2015), where the instructor needs to construct and convey information and the builder needs to comprehend it. The instructor could theoretically use an infinite number of words to ensure no ambiguous messages are sent. However, to avoid overwhelming listeners with information, instructors choose a fine-grained balance of words by presenting the right amount of information and adapted to their recipients. In this study, when little or no uncertainty emerged, instructors uttered non-fragmented instructions that were longer in duration than fragmented instructions, potentially leading to the conclusion that non-fragmented utterances could also have more efficient information compiled in a single utterance.

We predicted that *(1) speakers spend less cognitive resources in producing “one-shot” non-fragmented instructions, rather than fragmented instructions adapted to listeners*, assuming audience design costs more effort as the speaker needs to pause and adjust to the listener's signals of understanding. We indeed found in the data that instructors' cognitive load was higher in fragmented rather than non-fragmented instruction utterances, meaning that the role of the listener may have been a significant factor on speaker resources, and as such silent pauses and fragmented utterances appeared. Fragmented instructions seem to cause more effort for speakers, yet it is still unclear if that is due to the initial construction of non-efficient utterances or due to the listeners' misunderstanding^9^. This effect appeared to be more clear when looking at fragmented instructions in isolation. In every new fragment, the speaker required more resources to reduce uncertainty in attempts to reformulate previous fragments. We also found that overall fragmented utterances seemed to be shorter in duration in comparison to non-fragmented utterances indicating different levels of efficiency and precision to describe common objects.

It is also likely that these sequential turn expansions in fragments exist in order to convey messages in a less threatening manner. Presenting information in parts may reflect indirectness in conversation, in order to make a positive impression or maintain “face” (Goffman, 1955). It is therefore likely these adaptation strategies facilitate social coordination, even by presenting less efficient messages^10^. This indirectness may help to reduce the force of a message by not explicitly conveying the speaker's intent at once, and by helping into maintaining positive affect between the conversational partners^11^.

### 4.2. Effects of Listener Behaviour

In spontaneous human conversation, when a new utterance occurs, it remains ungrounded until the listener has showed positive evidence of understanding (Clark and Brennan, 1991). Continuous listener uncertainty however may cause the instructor to speak more turns in fragments until a satisfactory level of understanding has been reached. These states of uncertainty in conversation can be defined as binary classification tasks where according to listener uncertainty each new fragment is either grounded or ungrounded (Kontogiorgos et al., 2019), in what DeVault (2008) refers to as contribution tracking. Listener uncertainty may be expressed verbally, but the state of uncertainty in ungrounded fragments is most often expressed non-verbally (Kontogiorgos et al., 2019). Each speaker utterance is therefore planned as a collaborative action, where the speaker closely monitors the listener for understanding, and continuously reformulates utterances to resolve ambiguities. Following each other's eye-gaze through joint (shared) attention^12^ was the conversational partners contributions to mutual understanding, since conversation was concerned around objects.

Joint attention in this context was not different between non-fragmented and fragmented utterances. However, each new fragment appeared to cause convergence in joint attention, showing that speaker efforts were successfully met by the builders. Similarly, builders were faster at detecting^13^ referent objects in fragmented utterances, which also appeared to be decreasing in every new fragment spoken. Interestingly, we can see the instructors' concerns over being understood in each new fragment, gaze-to-builder appears to gradually increase, indicating instructors' continuous expectations for positive evidence of understanding. However, mutual gaze did not appear to differ in either non-fragmented or fragmented utterances.

We also predicted that *(2) speakers continuously attempt to reduce and minimise their listeners' cognitive load*, by producing instructions in fragments, and let the listener extract information incrementally. Pupillary responses demonstrated that this assumption was partially correct. We also found that builders' cognitive load was lower in fragmented instructions, perhaps indicating a preference in receiving information incrementally. Additionally, looking at fragmented utterances in isolation, we found that builders' pupil size decreased and that therefore instructor fragment and adaptation strategies were successful. The data indicated that each new fragment also caused an increase in instructors' cognitive load, showing that resolving ambiguities is a demanding task. This interplay of contributions to the task and mutual understanding, could mean that not both conversational partners show the same preferences in minimal and non-ambiguous instructions.

## 5. Implications for the Design of Conversational Interfaces

We found differences in preference for minimal and non-ambiguous instructions by instructor and builder participants, as shown in their behavioural metrics and indications of uncertainty. Fragmented instructions seemed to be associated with the listeners' signals of understanding and with the notions of adaptation. Conversational interfaces that instruct humans encounter various situations of listener uncertainty and when attentional expectations are not met, they should afford the flexibility of linguistic devices such as the construction of instructions in fragments. In this discussion, we aim to raise challenges that remain open, and yet to be empirically investigated, on how these results can be best applied to the design of artificial instructors.

### 5.1. Adaptation Strategies

Construction of minimal and non-ambiguous instructions may not be as important as adaptation strategies and adaptive design of instructions. Users may express signals of misunderstanding and uncertainty even in perfectly executed instructions, and may expect conversational interfaces to expand their instructions in fragments, such as human instructors do. Under the assumption that instructions are just conveying information, why should we invest time designing systems that utter “imperfect” or impartial instructions? There is the view that overall, conversation is not only about conveying information but also to perform a variety of social functions. Socially intelligent speakers in fact, construct imperfect speech with discourse markers, incomplete utterances, and disfluencies (Heeman and Allen, 1999). Eventually, social robots and conversational interfaces may not always need to attempt to transmit information as efficiently as possible, but instead replicate such human-like imperfections when they interact with humans. Rather than producing the absolute best and most efficient utterances, robots could focus on producing satisfying utterances for the design requirement and if understanding problems occur, attempt to clarify.

Listener adaptation may be an important robot skill, as robots move toward becoming efficient instructors. As Kiesler (2005) writes, the more robots become adaptive to their users, the less users may need to adapt to the robots. Research so far indicates that robots and conversational interfaces using flexible linguistic techniques increase user satisfaction over systems that employ “generic listener” methods (Pelikan and Broth, 2016; Foster, 2019).

### 5.2. Message Construction

In order to replicate the human flexibility in face-to-face communication, we need to computationally explain strategies of linguistic adaptation in message construction. While it may be more challenging for robots to always produce non-ambiguous instructions, constructing messages in fragments and letting the user participate in utterance production could convey human-like behaviour. Prior research has investigated message production strategies for instructional robots and has found that adapting messages to users' expertise largely affects user experience (Torrey et al., 2006). Grouping or summarising various instructional elements as adaptive strategies has also shown to affect the outcome of interactions with robot instructors (Sauppé and Mutlu, 2014). Research has also explored robots that incrementally ground uncertainty with human users (Hough and Schlangen, 2017), or dialogue systems that tend to be preferred when showing abilities such as rephrasing problematic parts of their utterances (Buschmeier et al., 2012).

In dialogue systems research, Zarrieß and Schlangen (2016) have found that referential success improves when providing information in installments. Other studies have also found that episodic generation of instructions from systems resembles human speakers when planning-based methods of sentence generation are used (Garoufi and Koller, 2014). Regarding adaptation strategies in message construction, some studies have investigated methods that track the listener's gaze to infer when to provide additional information on system instructions in installments (Koller et al., 2012; Staudte et al., 2012), showing that such adaptation strategies tend to be preferred by users. Similar research has investigated instruction generation strategies in systems that direct the user's attention to the referent when uncertainty is detected, also examining different types of feedback mechanisms, and have been perceived as being more cooperative (Mitev et al., 2018). Overall, dynamic and episodic construction of instructions seems to be perceived as a human-like behaviour, rather than non-ambiguous descriptions (Wallbridge et al., 2019), as this is not how humans naturally communicate instructions (Striegnitz et al., 2012). On the contrary, some prior research has also investigated systems that attempt to design the dialogue in a manner that users will not need to ask any questions, in order to avoid at any cost clarification and utterance reformulation (Bernsen et al., 1996). Such strategies in message construction also exist in modern dialogue systems, where conversations with smart-speakers are typically in the form of single-turn information retrieval tasks, with limited possibilities of flexible and adaptive behaviour.

We should nevertheless consider that there is a risk that humans will interact with robots that utter fragmented instructions differently than how they do with human instructors. There is evidence that users tend to vary their behaviour when they interact with computers, in comparison to how they interact with humans (Wu et al., 2020). It is therefore likely users would adapt to systems that provide too little or too much information, even when clearly violating the maxims of quantity (Grice, 1975), and when such behaviour from humans could appear to be odd. This prior work including our own findings, present opportunities for empirical investigations and user studies with robots instructing humans in installments. With a high control of isolating instructions into fragments, robots should monitor their listeners' cognitive load and attention, in order to expand their turns when listener behaviour indicates uncertainty. In an attempt to continuously minimise listener effort, experiments with human users should be designed to investigate what robot strategies are beneficial and what listener signals are informative to construct messages and instructions in fragments.

### 5.3. Design Issues

Using our findings on construction of fragmented instructions in human interactions, we discuss their applicability for designing instructions in human-robot collaboration. Many questions remain open on what interactive system designers should consider also when designing user studies and implementing instructional dialogue strategies^14^.

**How much information should a robot transmit in fragmented instructions?** We saw a variation in information transmission from human speakers, and overall, less time-efficient instructions were produced without fragments. However, empirical investigations of how much information should be transmitted are necessary, and nevertheless task-dependent. As long as robots can resolve uncertainty and are always ready to provide additional information, *they can initiate instructions that are incomplete or even ambiguous* in an attempt to not overwhelm users with information^15^. This means that *robots may need to consider withholding parts of the instruction from users*, by presenting the most important elements of the instructions first (Zarrieß and Schlangen, 2016), and expand their turns with fragments, if necessary^16^. Real-time incremental detection of user states of uncertainty will be necessary to decide when the robot should elaborate or reformulate instructions.

**When and how should a robot elaborate or reformulate previous utterances?** Withholding information in first attempts of instructions may display that the robot has trust on the user's understanding, and sequentially providing more information in fragments should convey collaborative behaviour. However, *timing instructions is crucial*. Variables such as pauses and fillers between fragments should be further investigated to determine appropriate timing between fragments. Too long pauses may indicate that the robot has finished its turn and passes the floor to the user, whereas short pauses may cause overlaps with the users' acknowledgments or actions. Enough space should therefore be considered for user feedback. In task-oriented dialogues user actions also represent turns (Galati, 2011). Robots should therefore attend to user actions before taking a decision to reformulate their instructions. In robot requests such as “can you pass me the salt,” a contingent compliance from the user should be expected and monitored before uttering “it is on your right,” “next to the pepper,” and so on. Each action is “conditionally relevant” and contributes to mutual understanding. Additionally, *what information in presented first may matter to users*. Some information may be more important to start instructions with (Zarrieß and Schlangen, 2016), while other elaborations may only need to be revealed when uncertainties arise^17^. What information attributes should fragments consist of is also task dependent and needs to be further investigated.

**What social cues should the robot attend to, in order to take decisions on new fragments?** The state of uncertainty is a complicated intrinsic state that may not always be expressed in users. There are display rules of expressions of emotions that regulate how people react in conversation (Ekman and Keltner, 1997), affective states such as uncertainty may not always be clearly expressed. Users may in some cases signal uncertainty with facial expressions or eye-gaze. In face-to-face task-oriented interactions eye-gaze in particular is a powerful predictor of confirmation or uncertainty. *Closely monitoring users' attention should indicate if the robot needs to utter more fragments in instructions*. Verbal clarifications are also common means of signalling feedback in interactions, however they require more effort from users and more turns need to be exchanged. A proactive attention-oriented fragmented utterance behaviour may be preferred (i.e., “to your left,” “top left”) (Mitev et al., 2018). Overall, the choice of robot embodiment, robot behaviour and sensory input may also affect how users behave and therefore what user information the robot will collect, influencing how the robot will convey additional fragmentary utterances to resolve ambiguities. Grounding in humans occurs with a number of verbal and non-verbal channels available, therefore what channels are available to robots will significantly affect their fragmented utterance production strategies.

## 6. Conclusions

### 6.1. Limitations

In this work we conducted a first investigation of cognitive resources allocation during the production of fragmented instructions in task-oriented dialogues. The constrained nature of the task provides an advantage in the systematic and controlled analysis of the interactions, however it also affects the ecological validity over other forms of conversation, i.e., open-world dialogues (Bohus and Horvitz, 2009), that are not concerned about objects, and where shared attention behaviours do not always apply. The states of uncertainty, ambiguity, and how speakers construct utterances in installments may differ in nature, in settings other than referential communication or where dialogue is non-collaborative. Our findings should therefore be interpreted cautiously when designing interaction paradigms under a variety of settings.

We made an attempt to avoid experimenter biases by using the Chinese Whispers experimental paradigm, yet subjects' lexical choices may have been restricted by the presented stimuli. There was little variability of non task-relevant objects in the visual scene which should also encourage discussion toward the generalisation of the findings to daily task-oriented settings, along with the distribution of rigid participant roles in the interaction. Moreover, in this experiment, we had little control of the conversational stimuli (fragmented utterances), which further indicates the importance of controlled experiments that measure responses to fragmented instructions in non-spontaneous dialogue.

Additionally, even though participants attribute little to no interference of sensory equipment in the task (eye-tracking glasses, motion-capture sensors, and microphones), there may have been an effect on their non-verbal behaviour that further places the interactions in a restrictive setting. While it is hard to predict what input future non-intrusive sensory equipment will have, we are also restricted by required data on pupillary responses. Eye-tracking devices are useful tools for analysis and experimentation, but are non-ubiquitous sensors that cannot currently be placed in state-of-the-art conversational interfaces. Access to users' pupil diameter in real-world settings is therefore challenging and also sensitive to external factors such as light conditions. In this setting, we ensured there is no interference by external factors to the pupil, by controlling for constant illumination in the experiment room, and by filtering the pupil signal to sudden changes (i.e., blinks or saccades).

Finally, all signals in this study were extracted automatically using sensory equipment, therefore some interactions had to be excluded due to missing sensory data, and we are bound to sensor quality available at the time the data was collected in spring 2018. While automatic annotation has benefits in efforts collecting the data, it should be noted that utterance coding had to be performed manually. Additionally, a lot of multimodal information is still underrepresented, either due to limitations in sensory equipment, or data not captured in this experiment such as facial expressions.

### 6.2. Concluding Remarks

The current study demonstrated that pupillary responses can be useful to examine the production of speech in fragments, by analysing group coordination but also each speaker in isolation. We also verified through the use of pupillary data what previous studies have indicated, that cognitive load increases through the increased use of pauses. Although these findings should be interpreted with caution, they do suggest that the fragmented production of instructions has both an effect on how information is exchanged within the principles of audience design and least-collaborative-effort, but may also be used as a tool for resolving ambiguities in dialogue. We predicted that speakers attempt to minimise listeners' cognitive load with the use of fragmented instructions. While this was partially true, the data indicated that fragmented instructions also cost more cognitive effort for speakers. A fine-grained balance of efficiently constructed instructions and adaptive behaviour by elaborating and reformulating instructions may therefore be necessary.

To conclude, while efficiently formulated instructions may minimise ambiguities, social robots and conversational interfaces that instruct humans may always need to be prepared for resolving ambiguities with the use of fragmented utterances. Repairing mismatches in common ground, robots following fragmented instruction strategies present an opportunity for adaptive behaviour and positive affect between the conversational partners. It should be noted that while we have raised open challenges to how these findings can be applied to human-robot interactions, such settings still need to be empirically examined. Finally, we discussed in this section conversational strategies for instructional robots, however we have not focused on conversational interfaces that respond to user fragmented utterances such as the work of Bell et al. (2001). Future interfaces should be able to handle the production of fragmented utterances in both the role of the speaker or the listener.

## Data Availability Statement

Publicly available datasets were analysed in this study. This data can be found at: https://www.kth.se/profile/diko/page/material and https://zenodo.org/record/4587308.

## Ethics Statement

Ethical review and approval was not required for the study on human participants in accordance with the local legislation and institutional requirements. The patients/participants provided their written informed consent to participate in this study. Written informed consent was obtained from the individual(s) for the publication of any potentially identifiable images included in this article.

## Author Contributions

The work reported here is part of DK's Ph.D. research under the supervision of JG. DK and JG designed the research and posed the research questions. DK collected and analysed the data. DK and JG wrote and edited the manuscript. Both authors have made direct and intellectual contribution to the work, and approved it for publication.

## Conflict of Interest

The authors declare that the research was conducted in the absence of any commercial or financial relationships that could be construed as a potential conflict of interest.

## Footnotes

^1^Also known as audience (or recipient) design.

^2^For reviews in pupillometry see: Kahneman and Beatty (1966), Beatty (1982), Beatty et al. (2000), Laeng et al. (2012), and Sirois and Brisson (2014).

^3^The notion of fragments has also been used in different contexts by Schlangen (2003) to refer to non-sentential utterances. Here we use a different definition of fragment, closer to the definition of Clark's installments.

^4^Fragments are coded similarly to Bell et al. (2001). We follow the notion of utterance from Meteer et al. (1995), sentences that can be coded into smaller units (“slash units”), parts of the instruction narrative interpreted as complete.

^5^Dialogue 1 for a sample of a fragmented instruction, Dialogue 2 for a sample of a non-fragmented instruction.

^6^4 instruction units with duration more than 20 s were excluded from the analysis.

^7^4 instructions had also a 5th fragment which we exclude due to the small number of occurrences.

^8^The forward-backward method from Gustafsson (1996) was used in the filter.

^9^Since listeners are present during utterance construction, speakers may interrupt themselves mid-utterance and reformulate their messages according to what the listeners do (Clark and Wilkes-Gibbs, 1986). Listener actions also count as turns, even when they are performed without speaking (Galati, 2011).

^10^For an extended discussion on how people tune messages to their conversational partners refer to Communication Accommodation Theory (CAT) (Gallois and Giles, 2015).

^11^We would like to point to the reader that the notion of indirectness presented here is not meant to be associated with indirect speech acts (Searle, 1975).

^12^Shared attention is one of the fundamental interactional phenomena in face-to-face conversation, where speakers coordinate actions and intentions by following each other's gaze (Tomasello et al., 2005).

^13^A reference resolution system developed in Kontogiorgos et al. (2018), found that instructors' speech and attention were the most prominent signals to intent recognition in collaborative assembly tasks.

^14^An overview of the questions in this article has first been presented in Kontogiorgos and Pelikan (2020).

^15^A risk exists that robots always starting with ambiguous instructions may lead users to lose trust to the system's capabilities.

^16^It is possible by the time the robot has decided to issue more information with a fragment, ambiguities have already been resolved by the user, and the additional robot turn serves the purpose of confirmation (just like backchannels) rather than disambiguation. We noticed similar behaviour between humans in this corpus (see dialogue sample 1, Utt-4).

^17^For elaboration theory refer to Reigeluth et al. (1980).
